# A Global Perspective of *GBA1*-Related Parkinson’s Disease: A Narrative Review

**DOI:** 10.3390/genes15121605

**Published:** 2024-12-16

**Authors:** Christos Koros, Anastasia Bougea, Ioanna Alefanti, Athina Maria Simitsi, Nikolaos Papagiannakis, Ioanna Pachi, Evangelos Sfikas, Roubina Antonelou, Leonidas Stefanis

**Affiliations:** 1st Department of Neurology, Eginition Hospital, National and Kapodistrian University of Athens, 11528 Athens, Greece; chkoros@gmail.com (C.K.); jalefanti@gmail.com (I.A.); simitch@yahoo.gr (A.M.S.); nickpap88@gmail.com (N.P.); ionpachi@hotmail.com (I.P.); vasfikas@gmail.com (E.S.); rantonelou@gmail.com (R.A.); lstefanis@med.uoa.gr (L.S.)

**Keywords:** Parkinson’s disease, genetic, glucocerebrosidase, GBA1, ethnic differences

## Abstract

Parkinson’s disease (PD) is considered to be the second most prominent neurodegenerative disease and has a global prevalence. Glucocerebrosidase (*GBA1*) gene mutations represent a significant hereditary risk factor for the development of PD and have a profound impact on the motor and cognitive progression of the disease. The aim of this review is to summarize the literature data on the prevalence, type, and peculiarities of *GBA1* mutations in populations of different ethnic backgrounds. We reviewed articles spanning the 2000–2024 period. *GBA1*-related PD has a worldwide distribution. It has long been recognized that pathogenic *GBA1* mutations are particularly common in certain ethnic populations, including PD patients of Ashkenazi Jewish ancestry. Moreover, a considerable number of studies focused on European ancestry patients from Europe and North America have revealed a high proportion (up to 15%) of carriers among the PD population. *GBA1* mutations also appear to play an important role in patient groups with an East Asian background, although the frequency of specific variants may differ as compared to those of European ancestry. Notably, the assessment of underrepresented populations in other parts of Asia (including India) and Latin America is in the spotlight of current research, while a variant with a newly described pathogenic mechanism has been reported in Sub-Saharan Africans. Given the importance of *GBA1* mutations for PD genetics and clinical phenotype, a focused assessment of the prevalence and type of *GBA1* variants in distinct ethnic populations will possibly inform ongoing PD-related clinical studies and facilitate upcoming therapeutic trials.

## 1. Introduction

Parkinson’s disease (PD) represents the second most frequent neurodegenerative disorder. Despite the fact that the majority of cases are idiopathic, PD has a strong genetic component, especially in cases of early onset (disease onset before 40 years of age) or familial inheritance. Approximately 15% of patients harbor pathogenic or likely pathogenic mutations in genes linked to PD [α-synuclein (*SNCA*), leucine-rich repeat kinase 2 (*LRRK2*), Parkin (*PRKN*), PTEN induced kinase 1 (*PINK1*), Parkinson’s disease protein 7 (*DJ-1*)] or genetic risk factors like the glucocerebrosidase gene (*GBA1*).

Pathogenic mutations in genes with an autosomal dominant inheritance may resemble typical idiopathic PD (*LRRK2*) or could result in an early-onset phenotype with pronounced motor and non-motor manifestations and a rapid disease course, as is often the case for *SNCA* mutation carriers. In contrast, pathogenic mutations in autosomal recessive genes like *PRKN* or *PINK1* usually result in a relatively mild phenotype but patients are susceptible to the appearance of early motor complications (fluctuations and dyskinesias) following treatment with Levodopa. Despite the fact that most cases have a positive family history, certain patients may seem apparently sporadic. Moreover, polymorphisms in common genetic variants including *SNCA* might slightly increase an individual’s risk of developing PD in later life and there are also polygenic forms as a result of simultaneously carrying multiple variants with minor risk effects. Genetic forms of PD have a variable phenotype, penetrance, inheritance pattern, and prognosis, therefore the genetic assessment is deemed to be increasingly important during the last few years for the additional reason that it may possibly impact the optimal therapeutic handling of each PD patient. There are accumulating data regarding the differential response of genetic PD forms to device-aided treatments [like Deep Brain Stimulation (DBS) or Levodopa–Carbidopa enteric gel pumps]. In addition, in the context of precision medicine, ongoing clinical studies may recruit only carriers of mutations of a specific gene, including *GBA1*. Novel putative disease-modifying therapies for PD targeting different molecular pathways are in the pipeline. Such therapies including exosomes carrying therapeutic compounds, small interfering RNAs (siRNAs), or micro RNAs (miRNAs) might also be relevant to the genetic background [1,2].

*GBA1* gene mutations are considered to be the commonest hereditary predisposing (risk) factor for PD. Glucocerebrosidase (GCase) (the translation product of the *GBA1* gene) is a lysosomal enzyme that decomposes glucosylceramide into glucose and ceramide [3]. More than 500 *GBA1* mutations have been described. Such mutations result in a marked loss of glucocerebrosidase enzyme activity. Single nucleotide variants, splice site variants, small deletions/insertions, wide deletions/duplications, and complex rearrangements involving *GBA1* and its nearby pseudogene *GBAP1* have been reported as pathogenic *GBA1* mutations. Homozygotes or combined heterozygote carriers of *GBA1* mutations result in Gaucher’s Disease (GB), a lysosomal storage disorder with an autosomal recessive form of inheritance. The most common form of GD is non-neuronopathic type I. In contrast, neuronopathic forms (GD type II and III) have an impact on the CNS (central nervous system). GBA1 pathogenic variants can be further categorized as either mild or severe depending on whether they predispose to neuropathic forms of GD or not. GD patients are at increased risk of developing Parkinsonian symptoms, and this eventually led to the clinical observation that even carriers of heterozygous pathogenic *GBA1* mutations are prone to manifesting PD [4,5,6,7]. The odds ratio for PD in *GBA1* carriers is approximately 5 to 6 [7]. Although *GBA1*-related PD has been observed in cases of familial inheritance (autosomal dominant pattern with reduced penetrance), many cases of patients harboring *GBA1* variants are apparently sporadic [8,9,10]. In addition, *GBA1* mutations are also the most important hereditary risk factor linked to Dementia with Lewy Bodies (DLB). Geiger et al. (2016) evaluated the genetic background of 111 DLB patients with an abnormal autopsy from North America and identified 13% to carry a disease-causing GBA1 mutation.

The pathway leading to PD in *GBA1* mutation carriers still remains elusive. Proposed mechanisms of causing PD include the accumulation of α-synuclein in neurons via a glucocerebrosidase–α-synuclein loop interaction. Autopsy studies in *GBA1*-related PD cases favor this option, as a widespread α-synuclein pathology has been witnessed. Other putative, possibly intersecting pathways involve lysosomal dysfunction and endoplasmic reticulum-associated stress. It remains to be shown whether a gain or loss of function is responsible for the detrimental effect, although most findings suggest the former [11].

Although *GBA1* is the most important predisposing factor for genetic PD and it has a global distribution, there are differences among various ethnic groups [12,13]. Most previous genetic studies have focused on patients of European background in Europe and North America. However, recent reports have underlined the importance of *GBA1* variants in other populations including East Asians and notably in underrepresented populations in Sub-Saharan Africa or Latin America.

PD patients carrying pathogenic *GBA1* mutations overall resemble idiopathic PD but exhibit certain distinct motor and non-motor symptoms [14,15,16]. An earlier onset age of 1.7–6.0 years has been described in *GBA1*-PD as compared to non-carriers [3,7,17]. In studies assessing early-onset PD patients, *GBA1* mutations were by far more common than in late-onset cases [18,19]. A prospective 3-year longitudinal study by Brockmann et al. [20] showed that PD-*GBA1* carriers, despite their earlier disease initiation as compared to non-carriers, had an overall worse disease course in terms of motor impairment. The worst disease course was mostly evident after the first few years of the disease. In the report of Sidransky et al., the motor outcomes of *GBA1*-PD and non-carriers were similar, although mutation carriers manifested a more symmetric onset [7]. An early development of motor complications in *GBA1*-PD has also been reported [21].

Furthermore, long-term assessment and analyses showed a worse trajectory of cognitive impairment in *GBA1*- PD patients. A higher prevalence of mental deterioration in *GBA1*-PD was also described in the large multicenter analysis of Sidransky [7]. *GBA1*-PD patients with EOPD performed worse in cognition assessment tests like MMSE in comparison to age-matched non-carriers and fulfilled diagnostic criteria for dementia more frequently [22]. Brockmann et al. also reported that the score of *GBA1*-PD patients when undergoing assessment using the MoCA test was inferior as compared to matched controls [20]. These results have also been verified by other large studies [23]. As far as detailed cognitive function domains are taken into account, *GBA1* carriers may have a rather marked deterioration in visual memory tasks, and this specific deficit may enhance their clinical detection [24].

Psychosis and other psychiatric symptoms may also be more frequent in *GBA1* carriers. Visual hallucinations appear to be present in up to 45.16% of *GBA1* carrier PD patients in a study in the British population [8]. In the study of Oeda et al., PD patients with *GBA1* mutations manifested psychosis more often and at younger ages than non-carriers [25]. Apart from psychotic symptoms, this group also exhibited more frequent depression, anxiety, apathy, and indifference compared to PD non-carriers (men were more susceptible) [26,27].

Finally, the frequency of other non-motor symptoms including hyposmia, REM sleep behavior disorder (RBD), and dysautonomia appear to be equally or even more prominent than in idiopathic PD [26]. In many previous studies, RBD appears to be more common in *GBA1*-PD patients compared to iPD [28,29]. Autonomic deficits (bowel function, orthostatic hypotension symptoms, urinary and sexual function) had an increased severity in carriers of *GBA1* mutations compared to non-carriers [26]. Notably, olfactory deficits in *GBA1*-PD could be more pronounced than in idiopathic PD, although the literature data are still limited [6,26,30].

Phenotype heterogeneity has also been shown to be linked to the type of *GBA1* mutation. It appears that severe mutations like L444P (or p.L483P according to the new nomenclature), c.1342G > C (D409H), c.115 + 1G >A (IVS2 + 1), c.84dupG (84GG), and c.1297G > T (V394L) result in an increased PD risk, disease onset at a younger age, and more devastating deficits in cognition compared to mild variants, like N370S (or p.N409S according to the new nomenclature) and c.84dupG (84GG) [31]. This was also true in the study by Cilia et al. [23]. Carriers of severe variants were more susceptible to dementia than those harboring mild ones. The type of these mutations differs according to the population assessed: N370S/N409S, c.84dupG (84GG), and c.1604G > A (R496H) are more frequent in Ashkenazi Jewish patients, while c.1342G > C (D409H), c.882T > G (H255Q), c.1093G > A (E326K), and c.1226A < G (N370S) can be found in European or West Asian patients. Furthermore, c. 475 C > T (R120W) is specific to East Asian populations and K198E has been described in some Latin American populations like Colombians [32].

The aim of this review is to summarize the literature data on the prevalence and type of *GBA1* mutations in populations of different ethnic backgrounds.

## 2. Materials and Methods

We reviewed articles published during the 2000–2024 period, focusing on genetic data in PD patients with a worldwide distribution. We included more studied populations in Europe and North America including Ashkenazi Jewish, Middle Eastern, East and Central Asian, Indian, Latin American, Sub-Saharan African, Oceanian, and Pacific Islands. We searched PUBMED, SCOPUS, and EMBASE from 2000 to 2024 using the following terms: “Parkinson’s disease”, “genetic”, “glucocerebrosidase gene”, “*GBA1*”, “Europe”, “North America”, “Ashkenazi Jewish”, “Middle East”, “East Asia”, “South East Asia”, “Central Asia”, “India”, “North Africa”, “Sub-Saharan Africa”, “Latin America”, “Oceania”, and “Pacific Islands”. Our literature search focused on genetic studies assessing *GBA1* carriers in monogenic forms of PD. Only articles published in English have been included in our present review.

## 3. Results

### 3.1. GBA1 in Ashkenazi Jews

Despite the global distribution of *GBA1*-associated PD, the genetic disease seems to be particularly prevalent amongst Ashkenazi Jews, where *GBA1* variants account for up to 15–20% of PD cases, with the N370S/N409S variant being the most common. A 2011 GWAS study in a highly homogeneous United States Ashkenazi Jewish population identified *LRRK2* and *GBA1* as the most prevalent PD susceptibility genes in this particular population [33].

A study by Shiner and colleagues evaluated the effect of *GBA1* variants on the clinical characteristics and progression of Dementia with Lewy Bodies (DLB) patients in a 35-patient Ashkenazi Jewish cohort. Among the patients, 11 out of 35 (31%) harbored pathogenic variants in the *GBA1* gene. Carriers exhibited an earlier age of symptom onset (65.7 vs. 72.1), scored higher on the RBD questionnaire, and had poorer cognition performance on the Montreal Cognitive Assessment Battery, with deficits on visuospatial function and verbal fluency tests. Moreover, higher mean scores on the Unified Parkinson’s Disease Rating Scale (UPDRS) part III indicated a greater degree of motor dysfunction. Overall, *GBA1* pathogenic variants have been linked to more severe motor and cognitive impairment [34]. Interestingly, carriers of both a mild *GBA1* mutation and the *APOE* ɛ4 allele were found to manifest more serious cognitive and motor dysfunction [35].

In another large study cohort of 1050 PD patients of Ashkenazi Jewish ancestry, 12 were determined to be either homozygotes or compound heterozygotes for pathogenic variants in the *GBA1* gene. They appeared to have more severe motor and non-motor symptoms (cognition, olfaction, RBD, and hallucinations) as compared to single mutation carriers. The authors conclude that the burden of *GBA1* variants with GD-PD is related to the severity of the PD phenotype [36].

The E326K variant has been demonstrated to raise the risk for PD, even if it is not typically classified as a Gaucher’s disease pathogenic variant. In a cohort of 1200 Ashkenazi Jewish PD patients, genotyping for 10 *GBA1* polymorphisms, *LRRK2*-G2019S and *SMPD1*-L302P revealed that E326K *GBA1* notably increased the risk for PD when co-occurring with another mutation [37].

Another study group evaluated 236 Ashkenazi Jewish PD patients in four different groups based on their mutation profile: *LRRK2*-PD, *GBA1*, *GBA1-LRRK2*-PD, and mutation-negative PD (MNPD). While dementia, probable RBD, and psychosis were more prevalent in *GBA1*-PD, these manifestations appear to be less frequent for *LRRK2-GBA1*-PD. Probable REM sleep behavior disorder (RBD) had a considerably higher rate in *GBA1*-PD than in *LRRK2*-PD, whereas none of the *GBA1-LRRK2*-PD cases noted RBD. Psychosis was most common in *GBA1*-PD and least common in *LRRK2-GBA1*-PD. It is possible that the co-existence of the *LRRK2* p.G2019S variant may exert a protective effect among *GBA1* variant carriers [38].

Finally, a study in Israel and the North American Jewish population concluded that full *GBA1* sequencing was by far more successful (17.4%) in detecting *GBA1* variants as compared to targeted genotyping. This method revealed that *GBA1* variants were present in 18% of PD patients and 7.5% (OR = 2.7) of healthy controls. Moreover, the p.E326K variant was identified as the second most common PD-related *GBA1* variant in Ashkenazi Jews, occurring in 1.6% of PD patients in this sample [39].

### 3.2. European Populations

*GBA1* variants are rather common in the European PD population, but the heterogeneity of prevalence and the type of pathogenic mutations among different countries has not been adequately addressed. A large international study, Rostock Parkinson’s disease study (ROPAD), assessed a group of 12,580 PD patients from 16 countries [representing four broader geographical regions (Europe, the Middle East, and North and South America)] with a predominantly European ancestry background [27.0% positive family history, median age at onset (AAO) of 59 years] and reported that 15% of patients harbored likely pathogenic variants. *GBA1* risk variants were the most prominent genetic outcome, as they were found in 10.4% of the population tested [40].

A Spanish study analyzed a cohort of 117 unrelated patients with early disease onset utilizing a combination of a Next-Generation Sequencing panel of 17 genes previously linked to Parkinson’s disease and CNV screening. Two patients (2/117) were identified with two likely pathogenic variants in the *GBA1* gene (p.Ile347Thr and p.Ser403Asn) that had not been previously documented. Interestingly, this screening failed to detect the most frequent pathogenic variants L444P/L483P and N370S/N409S [41].

Another research group assessing a South Spanish population of 134 patients (28 early-onset PD) found *GBA1* mutations in 7 patients (18.9%) (E326K, N370S/N409S, D409H, L444P/L483P). In the late-onset PD subgroup of 97 patients, pathogenic mutations in *GBA1* were identified in 13 patients (13.4%) (E326K, T369M, N370S/N409S, D409H, L444P/L483P) [42].

A large study conducted in the Italian population determined the frequency of *GBA1*-related PD in Italy and correlated *GBA1* variants with both motor and non-motor symptoms and their temporal occurrence [43]. This study evaluated 874 PD patients. Thirty-six variants were detected in 14.3% (fourteen severe, five complex, four mild, and three risk alleles) of the cases, including 20.4% among those with early onset. As expected, N370S/N409S and L444P/L483P, either isolated or as part of recombinant alleles, were the commonest variants with a cumulative frequency of 47.2% (N370S/N409S:30/125, 24.0% and L444P/L483P: 29/125, 23.2%, respectively) within the *GBA1*-PD group. A third variant, E326K, was also common (16/125, 12.8%). A number of non-motor features appeared earlier and more frequently in patients with *GBA1*-PD. Patients with severe and complex *GBA1*-PD exhibited a significant symptom burden, with an elevated risk of hallucinations and cognitive impairment [43].

In a study from the Sicily population, 126 PD patients of Caucasian and Sicilian ancestry were assessed using Next-Generation Sequencing [44]. In total, four *GBA1* variants were found: three have been already associated with PD (p.N409S, p.L483P, and p.T408M) and one with Lewy-body dementia (p.H294Q).

Bonato et al. screened a population of 218 PD patients from Northeastern Italy [45]. Patients were tested based on age at onset, family history, and the presence of atypical features. In total, 20% of the cohort participants harbored pathogenic variants and 28/218 (12.8%) carried pathogenic variants in *GBA1*. Of the identified genetic variants, *GBA1* had the highest frequency (30/133 variants, found in 27% of the 103 patients carrying genetic variants). The most prevalent *GBA1* variants in this patient group were N370S/N409S (11 cases) and p.Asp448His (D409H) (five cases), followed by p.Leu483Pro (L444P/L483P) and p.Glu365Lys (E326K), among others. In this group, 70% of *GBA1*-PD patients experienced symptoms before age 50 (mean age of onset: 47.8 years); 25% of these subjects noted a family history of PD; isolated rest tremor was uncommon at onset. Compared to mutation-negative PD patients, *GBA1*-PD patients experienced a greater load of motor complications, dyskinesias, and sleep problems including RBD as well as cognitive disturbances. Regarding cognition, *GBA1*-PD patients demonstrated a significantly lower mean score on the MoCA scale and a higher prevalence of dementia compared to mutation-negative controls. There was a trend for hallucinations, ICD, and psychiatric disturbances to occur more frequently in *GBA1*-PD compared to the mutation-negative control group and idiopathic late-onset PD, but this was not statistically significant; advanced therapeutic interventions, including deep brain stimulation (DBS) and levodopa–carbidopa intestinal gel (LCIG) therapy, were more frequently required by this group [45].

In the Greek population, two ethnic cohorts of individuals with sporadic PD were screened for eight distinct *GBA1* pathogenic variants, which account for 87% of the variants found in Gaucher disease patients diagnosed in Greece. Cohort A (*n* = 100) was composed of individuals from the Thessaly region in Central Greece, while cohort B (*n* = 105) included participants from the greater Athens area. Matched controls (*n* = 206) from the same areas were included. Eleven carriers of *GBA1* variants were detected in cohort A (5/11:N370S/N409S, 2/11:L444P/L483P, 2/11: D409H;H255Q, 1/11:H255Q, 1/11D409H) compared to three in the control cohort (*n* = 105). In cohort B, 10 carriers of *GBA1* variants were found (4/10:L444P/L483P, 4/10:D409H;H255Q, 1/10:N370S/N409S, 1/10:IVS10-1G→A), compared to 4 in the control cohort (*n* = 101). The difference in the joint number of carriers identified in PD patients of these cohorts and controls was statistically significant (OR 3.24). Patients with *GBA1*-PD demonstrated an earlier disease onset than non-carriers [19]. Furthermore, in a later study, a cohort of familial cases from the same center in the Athens region was analyzed, and seven patients (10.3%) were identified with *GBA1* gene mutations [46]. Thus, *GBA1* variants were equally prevalent in the familial cohort examined in this study as they were in the cohort of sporadic cases from the same center previously mentioned. Finally, the same group compared 35 Greek *GBA1* carriers with 35 genetically unidentified PD patients and revealed more cognitive deficits and a higher prevalence of bilateral motor symptoms onset in carriers of *GBA1* variants [47].

Crosiers et al. used a comprehensive sequencing of all coding exons of the *GBA1* gene to assess the prevalence of *GBA1* variants in a Flanders-Belgian cohort of 266 PD patients and 536 healthy controls. Rare, heterozygous *GBA1* variants were detected in 12 PD patients (4.5%) and 2 controls (0.37%) (OR 12.16). Compared to non-carriers, PD carriers had a more severe UPDRS motor score and the occurrence of dementia was significantly predicted by the *GBA1* mutation status. Notably, there was no genetic correlation between PD and the common p.E326K and p.T369M *GBA1* variants [48].

A large-scale Dutch study recruited a cohort of 3402 Dutch PD patients and 655 controls and performed sequencing of the entire *GBA1* gene. The researchers reported that 15% of all patients had nonsynonymous *GBA1* variants vs. 6.4% of controls (OR 2.6), including 5 PD-related variants (9.3% in patients and 4.4% in controls) and 18 novel variants. Importantly, 2.4% of patients and 0.9% of controls carried the generally rare, complex allele p.D140H + p.E326K, indicating that there is a founder effect in the Dutch population [49].

A UK-based study (the Tracking Parkinson’s study) evaluated 1893 recently diagnosed PD patients (≤3.5 years) and identified known Gaucher disease-causing *GBA1* pathogenic variants in 2.5% of the patients (*n* = 48), nonsynonymous variants previously associated with PD in 6.2% of the patients (*n* = 117), and variants of unknown significance in 1.5% of the patients (*n* = 28). The most frequent pathogenic *GBA1* variant was L444P/L483P. Comparing patients with pathogenic *GBA1* variants to those without, the former were, on average, 5 years younger when the disease first manifested. Moreover, *GBA1* variant carriers were more likely to present with the postural instability gait difficulty phenotype compared with non-carriers and were at a more advanced Hoehn and Yahr stage after adjustment for age and disease duration compared with non-carriers. No significant difference in cognitive function was observed between carriers and non-carriers, possibly due to all patients being recently diagnosed, while the more rapid cognitive decline of *GBA1*-PD manifests later in the disease course [50].

Ran et al. have evaluated the Swedish population for mutations in *GBA1*. Three *GBA1* variants, E326K, N370S/N409S, and L444P/L483P, were screened in 1625 cases and 2025 control individuals. The authors found a significant association with the high effect size of the rare variant L444P/L483P with PD (OR 8.17), while the common variant E326K showed a modest significant association (OR 1.6). The rare variant N370S/N409S showed a trend of association. Most L444P/L483P carriers (68%) were found to reside in Northern Sweden, which is consistent with a higher prevalence of Gaucher’s disease in this part of the country [51].

Similarly, a Finnish group assessed 527 Finnish patients with early-onset PD, 325 patients with late-onset PD, and 403 population controls. The screening effort included known genetic risk variants in *GBA1*, *SMPD1, LRRK2, POLG1, CHCHD10*, and *MAPT*. The p.N370S/N409S and p.L444P/L483P variants in *GBA1* contributed to a relative risk of 3.8 in early-onset PD and 2.5 in late-onset PD [52].

In a cohort from Portugal with 61 patients with familial PD, the entire open reading frames and exon-intron boundaries of *LRRK2* and *GBA1* were sequenced, without revealing any *GBA1* mutations [53].

A recent study from Hungary assessed the *GBA1* gene in a cohort of 190 Hungarian PD patients with suspected genetic risk. The study group reported 29 GBA1 rare variant carriers. The E365K and T408M variants represented the majority of detected variants (22 out of 32) [54].

Finally, genetic analysis was performed through screening for the *LRRK2* G2019S mutation, *GBA1* mutations (L444P/L483P, N370S/N409S), and common *GBA1* variants (E326K, T369M) in 762 PD patients and 400 controls from Russia [55]. The frequency of the *LRRK2* G2019S mutation was 5.8% in familial and 0.5% in sporadic PD cases. The frequency of *GBA1* mutations (L444P/L483P, N370S/N409S) in PD patients was higher compared to controls (OR 6.9, particularly in patients with early-onset compared to late-onset PD (OR 3.90). The frequency of E326K and T369M was twice as high among PD patients than in controls (OR = 2.24). However, no relevant *GBA1* mutations were identified in a cohort of 28 early-onset PD patients. Overall, *LRRK2* and *GBA1* mutations were found to be common risk factors for PD in the Northwestern region of Russia.

### 3.3. North America Populations

A large multi-center, observational study, PD GENEration, provides genetic testing, results disclosure, and genetic counseling to participants with PD across the US (including Puerto Rico), Canada, and the Dominican Republic [56]. In this study, DNA samples of 8301 PD patients were analyzed by next-generation sequencing with targeted testing for seven established PD-related genes. Reportable variants in *GBA1* were identified in 7.7% of all participants, while this percentage increased to 10.1% with the inclusion of the c.1223C>T T369M *GBA1* risk variant. The most frequently identified reportable *GBA1* variants were the E365K/E326K risk variant, found in 3.7% of participants, followed by the N370S/N409S variant in 1.6% and the L444P/L483P variant in 0.8%. Notably, among the participants, 14 carried two *GBA1* variants, with 9 having homozygous variants, while 6 of these cases involved Gaucher disease-associated variants, including N370S/N409S [56].

A Canadian group sequenced all exons of the *GBA1* gene in 225 PD patients and 110 control individuals from Eastern Canada. They were able to detect five known *GBA1* mutations of R120W, N370S/N409S, L444P/L483P, RecNciI, and RecTL (del55/D409H/RecNciI); two novel *GBA1* variants (c.-119 A/G and S(-35)N); as well as two risk variants, E326K and T369M, in PD patients. In contrast, only one S13L mutation and the two risk variants of E326K and T369M were found in the control group (PD vs. control OR 4.835). The frequency of the *GBA1* mutations (excluding the novel and risk variants) was estimated at 4.4% of the PD patients. The most common mutations of N370S/N409S and L444P/L483P accounted for 36% (9/25) of all the *GBA1* mutations in this Eastern Canadian PD cohort. The frequency of the E326K and T369M (6.67%) variants in PD patients was comparable to that of controls, suggesting that these two variants are not linked to PD in this population and may not have a pathogenic role. Unlike other studies, no significant difference in family history, age at onset, and cognitive impairment was identified between PD-*GBA1* and PD non-carriers [57].

### 3.4. East Asian Populations

*GBA1* variants have been identified in over 5% of PD cases across several Asian populations. *GBA1*-related PD appears to be prevalent in the Chinese population. A recent Chinese study screened for *GBA1* variants in a large cohort of 4034 patients and 2931 control participants using whole-exome and whole-genome sequencing. They reported 104 *GBA1* variants, including 8 novel variants. The frequency of *GBA1* variants in patients with PD was 7.46%, manyfold higher than that in controls (1.81%) (OR 4.38) [58]. These results were in agreement with previous Chinese studies. Similarly, Yu et al. reported that the prevalence of *GBA1* variants in a group of 184 PD patients was 8.7%. Upon this screening, they identified three novel (5-bp deletion (c.334_338delCAGAA), L264I, and L314V) and nine already reported *GBA1* variants (R163Q, F213I, E326K, S364S, F347L, V375L, L444P/L483P, RecNciI, and Q497R) [59]. According to another Chinese study, the frequency of *GBA1* variants was 10.72% (79 out of 737 patients), with R163Q, L444P/L483P, and R120W being the most common *GBA1* variants [60]. Furthermore, a study tested 1676 familial PD (both autosomal-dominant ADPD and autosomal-recessive ARPD) cases and sporadic cases with early-onset PD (sEOPD) from mainland China by utilizing gene dosage analysis and whole-exome sequencing [61]. They detected six possibly pathogenic variants of *GBA1* in 6 ARPD cases (6 out of 192, 3.15%), 10 in 10 ADPD cases (10 out of 242, 4.13%), and 45 in the sEOPD cohort (45 out of 1242, 3.62%). Overall, 59 probands were found to carry the p.L444P/L483P variant (3.52%, 59 of 1676), while the p.N370S/N409S variant was absent in the cohort.

A large Japanese study of 534 PD patients and 544 controls reported 50 PD carriers of a *GBA1* variant (9.4%), with R120W and L444P/RecNciI being the most prevalent odds OR (28.0) [62]. Similarly, in the pivotal multicenter study of Sidransky et al., *GBA1* pathogenic mutations were present in 9.4% of PD and 0.37% of controls, resulting in a remarkable odds ratio (OR 25.4) [7].

A screening performed among the Korean population in a cohort of 277 PD patients and 291 controls, by sequencing all exons of *GBA1*, revealed five different pathogenic heterozygous *GBA1* mutations in nine PD cases (3.2%). The variants detected were: N188S, P201H, R257Q, S271G, and L444P/L483P, with R257Q being the most common mutation. No *GBA1* mutations were found in the control group (OR 20.6) [63].

In Malaysia, *GBA1* mutations, particularly L444P/L483P, were also found in PD patients. A study of 496 patients of multi-ethnic (Chinese, Malay, and Indian) origin detected 14 heterozygous *GBA1* variants in 25 patients (5%). The most prevalent variant was p.L444P/L483P, found in all groups along with newly described variants. However, common variants previously reported in Europe, p.E365K, p.T408M, and p.N370S/N409S, were not found [64].

In a Taiwanese study, 967 PD patients and 780 controls were recruited; 3.72% of PD patients carried a pathogenic *GBA1* mutation (27 L444P/L483P, 7 RecNciI, and 2 D409H). In contrast, heterozygous *GBA1* mutations were reported in two controls (0.26%) (one L444P and one RecNciI). Therefore, the L444P/L483P GBA1 mutation in particular is associated with early-onset PD in the Han Chinese population and represents an important risk factor for PD in this population [65].

In the Thai population, a study involving 108 EOPD patients and 100 PD with disease onset after age 50 found heterozygous *GBA1* variants in 24 patients (5%). Two controls (0.5%) also harbored *GBA1* pathogenic variants. Seven distinct *GBA1* missense variants were detected, including p.L444P/L483P, p.N386K, p.P428S, IVS2+1G > A, IVS9+3G > C, IVS10-9_10GT > AG, and c.1309delG. Notably, five of the variants were novel [66]. Moreover, a comparison between early-onset and normal-onset PD revealed that *GBA1* variants were more frequent in early-onset patients (OR = 4.64).

### 3.5. Indian Subcontinent

*GBA1* variants, mainly L444P/L483P and others, which are rare in Europeans, are relatively common in the Indian population. In their analysis of whole genome sequencing data from a cohort of 90 young-onset PD cases, Kukkle et al. showed 13 PD patients (14.4%) carrying *GBA1* variants, including E365K, L444P/L483P, R159Q, D448H, P454L, R502P, and R502H variants [67]. In a different study, conducted in eastern India, researchers detected the p.L444P/L483P variant in 9 PD patients by screening a cohort of 198 patients with typical PD and 136 patients with PD and dementia, as reported in [68]. Kumar and co-authors performed whole exome sequencing in 250 Indian PD patients. Despite the fact that 11 PD cases carried only rare risk variants in *GBA1*, gene level analysis showed a higher burden of rare *GBA1* variants compared to controls [69]. Moreover, another group screened a cohort of 100 South Indian PD patients for mutations in the *GBA1* gene and found two novel variants (IVS1 + 191G > C and IVS10 + 3G > A) and three already described variants [70].

In a more recent large genetic study in India, 230 patients (87.4% were early-onset PD) underwent exome sequencing. Twenty-three patients harbored 13 *GBA1* variants. Fifteen out of twenty-three patients had severe or null variants. The L444P/L483P mutation was the commonest in this group while the Caucasian variant N370S/N409S and polymorphism E365K were completely absent. Similarly to previous reports, heterozygous *GBA1* variant carriers, compared to their idiopathic PD counterparts, appeared to have earlier-onset PD, a more rapid progression in terms of non-motor symptoms, and a prevailing akinetic-rigid phenotype. The genotype and phenotypic spectrum in this study were overall comparable to findings in other East-Asian countries [71].

### 3.6. Central Asia Populations

There is a paucity of genetic PD studies concerning *GBA1* variants in Central Asian countries. Kaiyrzhanov et al. performed WES on 50 unrelated individuals with young-onset PD from Kazakhstan, but no pathogenic *GBA1* variants were reported in this cohort [72].

### 3.7. Latin American-Caribbean Populations

Latin American populations of indigenous or mixed origin have been reported to harbor different *GBA1* variants than those encountered in the populations of European ancestry, with the prevalence varying by country. A multicenter, retrospective study screened PD-related genes in a familial PD cohort of 324 participants from seven Latin American countries (Argentina, Brazil, Chile, Colombia, Honduras, Peru, and Uruguay) that are part of the Latin American Research Consortium on the Genetics of Parkinson’s disease (LARGE-PD) [73]. *GBA1* pathogenic variants, including W184R, K198E, G202R, N370S/N409S, and L444P/L483P, were identified in 25 individuals (7.7%).

The LARGE-PD study focusing on Colombian and Peruvian populations screened 602 PD patients and 319 controls by sequencing the whole *GBA1* gene coding region. The researchers revealed a greater prevalence of *GBA1* variant carriers among the PD patients group compared to that of controls (5.5% vs. 1.6%, OR 4.3). Interestingly, Colombian patients had a higher mutation frequency (9.9%) compared to Peruvian patients (4.2%), which is explained by the presence of the ethnicity-specific p.K198E *GBA1* variant exclusively in the Colombian cohort [74]. Similarly, a Brazilian study found pathogenic *GBA1* variants in 5.4% of early-onset PD cases (6 out of 110 patients) [75]. In a separate targeted screening of a Brazilian PD cohort, 2.8% of index cases (4 out of 141) tested positive for the most common *GBA1* mutations (L444P/L483P or N370S/N409S), three with the L444P/L483P variant (2.1%) and one with the N370S/N409S variant (0.7%) [76]. Interestingly, most Brazilian studies have not employed full sequencing of the GBA1 gene, suggesting a potential underestimation of *GBA1*-related pathogenic variants in the Brazilian population [77].

Finally, a study group performed genome-wide genotyping in 79 Latino PD patients, of which approximately 80% identified as Caribbean Latino. They reported one *GBA1* p.T408M and three *GBA1* p.N370S/N409S carriers of European ancestry as well as three *GBA1* p.L13R variants on an African background. Variant *GBA1* p.K237E, previously observed in Latinos, was absent in this cohort. A rare, functional *GBA1* p.S310G variant was found in one individual heterozygous for European/American Indian ancestry [78]. It appears that the frequency of *GBA1* is lower (4.17%) in Latin American studies as compared to European/North American cohorts, probably due to limited access to gene sequencing [79].

### 3.8. Middle East and North African Populations 

The *GBA1* variants implicated in PD pathogenesis seem to be rare in the Middle East and North Africa (AfrAbia) populations. A study conducted on individuals primarily from North Africa, mainly Algeria, identified three heterozygous *GBA1* mutations (K27R, R131C, N370S/N409S), one homozygous R131C mutation, and two complex alleles [L444P/E326K, RecNciI (A456P/V460V/L444P)] in nine patients with PD (9 of 194, 4.6%), while only one heterozygous D443N variant was found in the control group (1 of 177, 0.5%) [80]. A Tunisian study analyzed 33 familial PD cases by screening all 11 exons of the *GBA1* gene [81], revealing two novel variants (p.K26R and p.K186R). However, the pathogenicity of these two variants has not been sufficiently confirmed. Additionally, the authors screened a cohort of 395 PD patients and 372 controls to specifically assess the presence of the N370S/N409SGBA1 variant. This was found in one patient and three controls, suggesting that the frequency of this variant is lower in the AfrAbian population compared to Ashkenazi Jews and that it does not represent a risk factor for PD in such an ethnic background.

### 3.9. Sub-Saharan Africa Populations

In Black African populations, *GBA1* variants are infrequent but distinct compared to the common variants found in European populations. A South African study of 30 PD patients of Black ancestry found two carriers harboring *GBA1* pathogenic variants (p.R120W and p.R131L) upon sequencing all 11 *GBA1* exons [82]. The screening of a Nigerian cohort for *GBA1* variants showed six likely pathogenic variants exclusively in PD patients (p.W184R, p.L383PfsX3, and three p.L444P/L483P), which correspond to 6.5% of this PD cohort [83]. Recently, a novel risk factor found in intron 8 of the *GBA1* gene (rs3115534-G locus) came to light in a large genome-wide association study (GWAS) in people of African and African mixed ancestry, with and without PD (overall meta-analysis OR for risk of PD 1.58). This variant, which appears to be linked to reduced glucocerebrosidase activity, was observed in 39% of Sub-Saharan PD patients, and yet it has not been reported in Europeans. These findings suggest a novel potential mechanism for PD pathogenesis in African populations and underscores the importance of conducting genetic testing in ethnically diverse cohorts [84]. Interestingly, a more recent study reported that the newly identified non-coding *GBA1* rs3115534 risk variant is related to a high prevalence of REM sleep behavior disorder (RBD) symptoms in persons of Nigerian origin with PD [85].

### 3.10. Oceanian Populations

Studies assessing PD genetics in Oceania, with a mixed European/Asian/Aboriginal-Maori population, are scarce. Graham et al. evaluated a New Zealand PD cohort (229 PD and 50 HC) using a recently developed nanopore DNA sequencing method to analyze *GBA1* variants [86]. The study group reported 23 variants in 21 PD cases (9.2% of PD patients compared with 4% of HCs). The most common mutations found were p.E365K in twelve cases, p.T408 M/p.T369M in seven cases (one also harboring p.E365K/p.E326K), and p.N409S/N370S in one case. Researchers also described additional putative *GBA1* variants p.R78C, p.D179H, and c.335C > T (a novel variant that possibly impacts the splicing of GBA1 transcripts) [86].

We were unable to include studies on GBA1 prevalence/mutations in Australian cohorts but isolated screening efforts are active and a novel *GBA1* risk variant, p. W378R, has been described [87].

### 3.11. Pacific Islands

**Figure 1:**
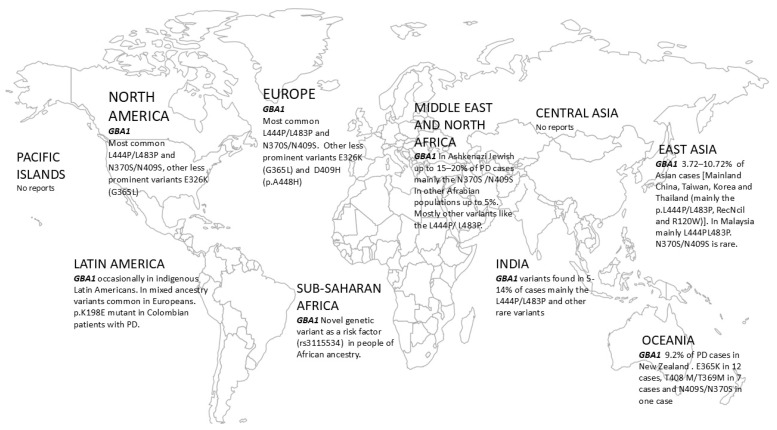
Distribution map of pathogenic GBA1 variants around the globe.

To our understanding, there are no studies assessing the frequency or type of *GBA1* variants in PD patients in the Pacific Islands, in spite of a few previous reports on other PD-related genes, including PINK1, in such populations. An overview of GBA1 variants and prevalence is shown in Table 1 and Figure 1.

## 4. Discussion

In the current review, we have assessed the prevalence and features of *GBA1*-related PD in populations of different ethnic groups across the globe. The importance of *GBA1* variants as the main predisposing factor for PD, with a worldwide prevalence, is increasingly recognized. We have to highlight the fact that there is a marked heterogeneity regarding the frequency and the type of *GBA1* variants in different ethnic or geographical groups. It is possible that at least in part it is due to the different screening methods employed across the studies. Environmental factors including diet differences or exposure to toxicants may further explain the diversity in the clinical phenotype of *GBA1* mutation carriers. *GBA1* pathogenic variants appear to be particularly common in certain populations, including patients of Ashkenazi Jewish descent. The mild variant N370S/N409S represents the main mutation found in this specific group. Most previous studies have taken place in populations of European backgrounds in Europe and/or North America. In European studies, the frequency of pathogenic *GBA1* variants in PD patients ranged from 5 to 15%, representing overall the most common hereditary risk factor. It has to be noted that even in certain European populations, such as in the Portuguese, some screening efforts failed to detect any carriers of *GBA1* mutations among PD patients, indicating heterogeneity even within the European continent. The varying methods employed in different studies, ranging from targeted approaches for a single variant to WES, may also contribute, and it is possible that the picture will become clearer when Next Generation Sequencing methods are more widely employed. The most commonly encountered variants are L444P/L483P and N370S/N409S in the majority of reports. A third variant, E326K, was also found in various European populations, including studies in Italy and Russia, while D409H was described mostly in a number of PD cases in Southern Spain and Greece. In North American studies, the frequency and variants of *GBA1* are similar to European studies, with a strong component of N370S/N409S, due to the Ashkenazi ancestry of a marked proportion of US and Canada residents with PD who have undergone genetic testing.

In East Asian populations, *GBA1* mutations were present in at least 5% of cases. N370S/N409S is rather rare when compared to European ancestry groups. In Mainland China, Taiwan, Korea, and Thailand the p.L444P/L483P, RecNcil, and R120W variants were mostly reported, while the L444P/L483P variant was reported in Malaysia. In the Indian subcontinent, *GBA1* variants, mainly L444P/L483P and other variants, some of which are rare in Europeans, were found in 5–14% of cases. Reports on *GBA1* variants in PD cohorts in Central Asia or the Pacific Islands are missing. In Latin American indigenous populations, *GBA1* variants were occasionally reported, whereas those of mixed ancestry harbored variants common in Europeans. In Middle Eastern and North African populations, the N370S/N409S variant appears to be rarer than in Ashkenazi Jews,, whereas other variants like L444P/L483P were occasionally described. Finally, in Sub-Saharan Africa, a novel genetic variant in *GBA1* has been recently discovered as a risk factor (present in 39% of cases). Overall, similarly to previous observations in patients of European ancestry, a worse phenotype of *GBA1*-PD patients has been described as compared to idiopathic PD in various non-European populations, and this is particularly true for carriers of severe mutations like p.L444P/L483P. A limitation of our current review is that there is some inconsistency in the samples of the sources used. For instance, studies comprising a few hundred patients are included alongside studies involving much larger groups, such as the Rostock Parkinson’s disease study, which encompasses over 12,000 patients. This could be attributed to the scarcity of genetic screening studies for certain populations including Sub-Saharan African or Central Asian patients. Genetic screening efforts in certain European countries are limited as well. Ongoing studies will further elucidate the phenotype of GBA1 carriers in different ethnic populations.

The fact that *GBA1* mutations or risk variants are very frequent in most populations is particularly significant for PD research. There has been a systematic bias in previous research on PD genetics, as most studies have assessed populations of European ancestry, mostly in Europe and North America [13]. Accumulating data from studies in East Asia (especially China) will possibly bridge the gap in genetic PD research. However, other ethnic groups still remain underrepresented. This is particularly true for other parts of Asia, the Middle East, Africa, and Latin America. Reasons for not including patients from these regions include the lack of an adequate number of trained health employees, limited resources, and the skepticism of patients enrolling in genetic studies due to their cultural or religious background. The effects of the geographical isolation of populations and genetic flow through admixture or migration need to be taken into account. Large screening efforts are underway in order to characterize diverse ethnic groups. *GBA1* variants are in the spotlight of such international studies. The Global Parkinson’s Genetics Program (GP2) is particularly important due to its worldwide reach [88]. The MJFF Global Genetic PD Cohort also expands the knowledge on the global PD genetic map [89]. Regional studies are also crucial, like the International Parkinson’s Disease Genomics Consortium Africa, the Latin American Research Consortium on the Genetics of Parkinson’s Disease, the AfrAbia-PD-Genetic Consortium (AAPDGC) and the GAP-India study [90,91,92,93].

## 5. Conclusions

A global perspective of pathogenic *GBA1* variants in PD is warranted due to ongoing clinical studies for disease-modifying treatments targeting the glucocerebrosidase pathway [93]. It is also clear that accumulating data on genetic PD forms will modify the algorithm of symptomatic treatment selection. Notably, *GBA1* carriers have been widely studied regarding their potentially differential response to device-aided therapy options, including deep brain stimulation and Levodopa–Carbidopa enteric gel or Apomorphine pumps [94,95]. A solid comprehension of the heterogeneity in *GBA1*-PD across populations will facilitate the inclusion of diverse populations in clinical trials, enhance our ability to reach new research milestones, and possibly pave the way for novel therapeutic targets in PD research.

## Figures and Tables

**Table 1:** Variation in GBA1 genetic background according to geographical/racial distribution.

Population	*GBA1*-PD Prevalence	*GBA1*-Variants
Ashkenazi Jewish	Up to 15–20% of PD cases.	Mainly the N370S/N409S variant. Other mutations include c.84dupG (84GG) and c.1604G > A (R496H).
European	5–15% of PD cases.	Most common L444P/L483P and N370S/N409S. Also, other less prominent variants are E326K (G365L) and D409H (p.A448H).
North American	Similar to European populations.	Most common variants are L444P/L483P, N370S/N409S, and E326K.
East Asian	*GBA1* pathogenic variants ranging between 3.72 and 10.72% in Asian PD cases.	In Mainland China, Taiwan, Korea, and Thailand, mainly p.L444P/L483P, RecNcil, and R120W. In Malaysia, mainly L444P/L483P. N370S/N409S is rare.
Indian	*GBA1* variants found in 5–14% of cases.	Mainly L444P/L483P and other variants (some rare in Europeans).
Middle Eastern and North African	Relatively rare (up to 4.6%).	N370S/N409S variant rather scarce compared to Ashkenazi Jews; other variants like the L444P/L483P occasionally described.
Sub-Saharan African	Common variants relatively rare but the rs3115534 variant is common as a risk factor (found in 39% of Sub-Saharan PD patients).	Novel genetic variant in *GBA1* (rs3115534) as a risk factor in people of African ancestry.
Latin American	Occasionally reported in indigenous Latin Americans, Similar to Europeans in patients of mixed ancestry.	Patients of mixed ancestry had mostly variants common in Europeans. p.K198E mutant in Colombian patients with PD.
Oceanian	9.2% of PD cases in New Zealand.	E365K in 12 cases, T408 M/T369M in 7 cases, and N409S/N370S in 1 case.

## Data Availability

No new data were created or analyzed in this study.
